# An Equivalent Magnetic-Circuit-Modeling Approach for Analysis of Conical Permanent Magnet Synchronous Motor

**DOI:** 10.3390/s25061788

**Published:** 2025-03-13

**Authors:** Fengrui Cui, Junquan Chen, Pengfei Hu, Xingyu Wu, Fangxu Sun

**Affiliations:** National Key Laboratory of Electromagnetic Energy, Naval University of Engineering, Wuhan 430033, China; 21000506@nue.edu.cn (F.C.); chenjunquan123@nue.edu.cn (J.C.); xingyu_wu@foxmail.com (X.W.); sunfx2013@hotmail.com (F.S.)

**Keywords:** rim-driven thruster (RDT), equivalent magnetic circuit model (EMCM), conical permanent magnet synchronous motor (CPMSM), lumped-parameter network modeling, finite-element analysis (FEA)

## Abstract

Shaftless propulsion technology delivers high efficiency and low noise for subsea installations and marine vessels. To enhance thrust performance, the streamlined aft-body contour imposes stringent demands on geometric compatibility between the rim-driven thruster (RDT) motor and hull. This necessitates advanced electromagnetic characterization of conical motors. This paper proposes an equivalent magnetic circuit model (EMCM) that accounts for end effects and magnetic saturation in both the stator and rotor cores for the magnetic field analysis of conical permanent magnet synchronous motor (CPMSM). A 3D EMCM is developed by decomposing the air-gap flux into radial/axial/tangential components. End-field nonlinearities are addressed via lumped-parameter network modeling. Innovatively, a trapezoidal expanded magnet layout and magnet-pole-trimming technology are adopted to ensure sinusoidal flux distribution. Finally, a 10.5 kW prototype with a conical angle of 6.7 degrees is designed using the EMCM and verified through a finite-element analysis (FEA) and experiments. This research provides a theoretical framework for the rapid electromagnetic analysis of the CPMSM.

## 1. Introduction

In recent years, industrial advancements have driven motor systems toward highly integrated development, paving the way for the emergence of shaftless rim-driven thrusters (RDTs) [1]. Building upon traditional electric propulsion systems, RDTs relocate the propulsion motor outside the hull and integrate it with the thruster, offering advantages such as high power density, low radiated noise, high propulsion efficiency, compact size, and lightweight construction. These features have established RDTs as the next-generation propulsion solution for underwater vehicles, including submarines [2,3,4,5].

The selection of propulsion motors remains a critical challenge in shaftless RDT technology, directly determining a vehicle’s navigational performance [6,7,8,9]. Permanent magnet synchronous motors (PMSMs) have emerged as an ideal choice for such applications due to their compact size, lightweight design, high power factor, superior efficiency, exceptional power density, simplified structure, sinusoidal air-gap magnetic field distribution, and low-vibration/noise characteristics [10,11,12,13].

However, as shaftless propulsion technology evolves, expanding the operational envelope of RDTs has become a focal research area [14,15,16,17]. To address this, this study proposes a conical permanent magnet synchronous motor (CPMSM) as a novel propulsion motor for shaftless systems. The design prioritizes geometric compatibility between the motor’s profile and the streamlined aft-body contours of marine vessels, aiming to optimize hydrodynamic integration and thrust performance.

CPMSMs in the traditional configuration feature conical-shaped stator and rotor, utilizing axial displacement to modulate air gap length. This inherent self-regulating excitation characteristic enables facile adjustments of no-load back electromotive force, axial magnetic forces, inductance, and torque performance parameters [18,19]. Consequently, conical motors are primarily deployed in high-speed turbine generation [20], electric vehicle drivetrains [21], and aerospace applications [22,23]. However, when applied as propulsion motors in shaftless RDT systems—where exceptional driving precision and vibration suppression are paramount—CPMSMs present critical challenges:

1. Axial Electromagnetic Force Interference: Structural inevitability of residual axial electromagnetic forces adversely affects thrust precision while introducing parasitic axial vibration sources.
2. End Leakage Flux Limitations: Geometric constraints of conical topology exacerbate magnetic flux leakage at extremities, constraining peak thrust output capability.

Thus, rapid and accurate electromagnetic performance analysis becomes pivotal in determining CPMSM suitability for RDT applications.

The Equivalent Magnetic Circuit Model (EMCM) is a widely adopted technique for motor analysis and design by correlating material properties with electromechanical behavior [24,25,26]. By applying circuit principles such as Kirchhoff’s Voltage Law (KVL) and Kirchhoff’s Current Law (KCL), magnetic field characteristics can be quantitatively resolved [27,28].

Traditional electromagnetic analysis models for CPMSMs predominantly employ 3D finite-element analysis (FEA) and Segmented Equivalent Magnetic Circuit Methods [18,21,29]. While offering high magnetic field calculation accuracy, the 3D FEA method incurs significant computational time consumption and lacks physical interpretability, rendering it unsuitable for rapid preliminary CPMSM design. The segmented magnetic circuit approach divides the CPMSM’s magnetic circuit into axial segments. By appropriately selecting segment lengths and assuming uniform magnetic fields within each cross-section, this method reduces 3D field analysis to multiple 2D field computations, with results aggregated to derive motor parameters. However, conventional 2D field calculations fail to account for the axial magnetic field distribution inherent to CPMSMs, and their accuracy in modeling end-region leakage fields remains unsatisfactory.

To address these limitations, this study proposes a generalized, high-fidelity, and computationally efficient EMCM tailored for CPMSM design and analysis, incorporating end-effect compensation and flux saturation considerations. The paper is structured as follows:

Section 2 elucidates the geometric compatibility of CPMSMs in shaftless propulsion systems and details their topological configuration.

Section 3 introduces the application of EMCM to CPMSM field analysis. A 3D EMCM is developed by decomposing the air-gap magnetic field into radial, axial, and tangential components. End-region field distribution patterns are analytically derived, and lumped-parameter network modeling are employed to address nonlinearities in end-region fields, effectively reducing 3D magnetic circuit problems to 2D solutions.

Section 4 analyzes the electromagnetic characteristics of CPMSMs using the proposed model. Innovations include a trapezoidal magnet arrangement and pole-arc trimming technology, ensuring consistent pole-arc coefficients across axial sections and sinusoidal air-gap fields. These advancements effectively mitigate interpolar leakage flux, cogging torque, load torque ripple, and axial force fluctuations.

Section 5 validates the methodology through a 10.5 kW prototype with a 6.7-degree cone angle, employing FEA and experimental verification. Comparative results demonstrate the model’s computational efficiency and predictive accuracy.

## 2. The Application of CPMSM in Shaftless RDT

### 2.1. The Geometric Compatibility of CPMSM in Shaftless RDT

**Figure 1:**
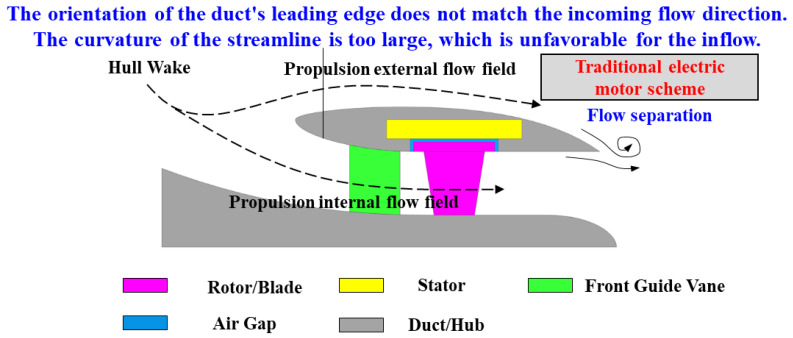
Traditional electric motor scheme in shaftless RDT.

In conventional shaftless RDT, the layout of traditional motors and duct designs often exhibit inherent limitations that compromise propulsion performance. As shown in Figure 1, when the motor orientation mismatches the incoming flow direction—requiring the duct’s flow-directing orientation to simultaneously align with hydrodynamic streams and accommodate the thruster motor—the following critical issues arise:

Duct Leading Edge: The stator’s aerodynamic compatibility necessitates a forward-oriented leading edge, resulting in poor synchronization with hull wake flows. This configuration disrupts inflow uniformity and impedes hydrodynamic optimization.

Duct Trailing Edge: Structural accommodation of the stator forces abrupt contraction at the trailing edge, inducing flow separation that increases drag and undermines low-noise design objectives.

Overall Duct Profile: Excessive volumetric footprint, elevated structural mass, and suboptimal streamline geometry collectively constrain RDT performance enhancement.

**Figure 2:**
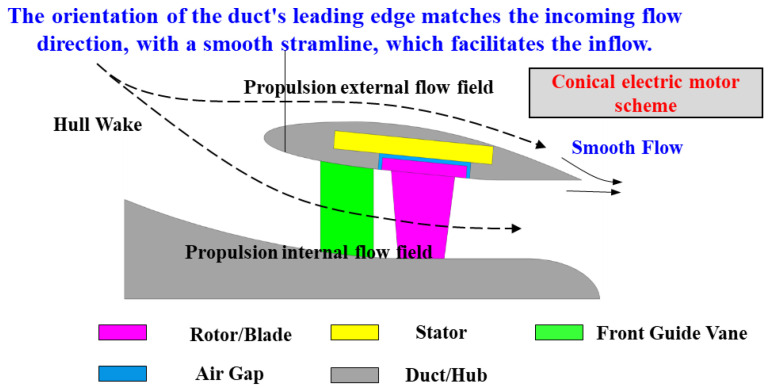
CPMSM scheme in shaftless RDT.

This study proposes a CPMSM configuration for RDTs, as illustrated in Figure 2. By reconfiguring the motor’s topological geometry, this solution achieves the following:

Enhanced Flow-Hull Synergy: Improved alignment between inflow patterns and hull wake fields facilitates integrated “hull-propulsor” co-design for hydrodynamic synergy.

Optimized Duct Aerodynamics: Streamlined duct contours reduce while suppressing flow separation vortices.

Compact Lightweight Design: Significantly improving system efficiency and maneuverability.

The CPMSM-enabled RDT architecture amplifies the intrinsic advantages of shaftless propulsion—high-efficiency, low-noise characteristics—while pioneering new methodologies for next-generation marine propulsion systems.

### 2.2. The Topological Structure of CPMSM

**Figure 3:**
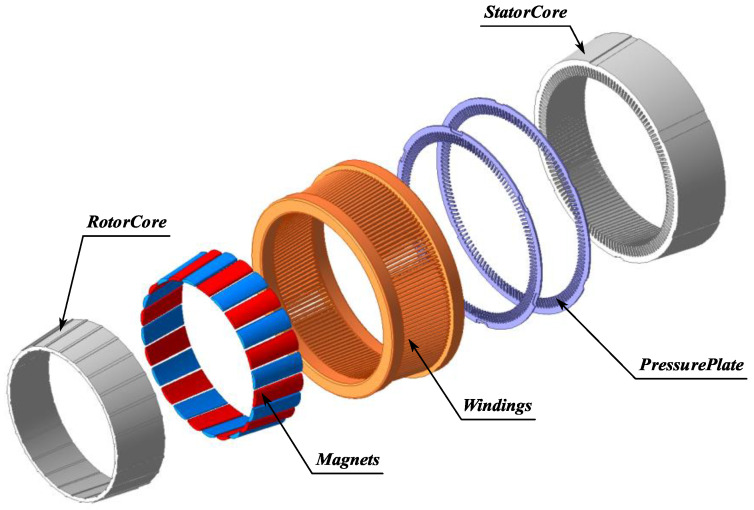
The topological structure of CPMSM.

In this paper, the proposed topology of the CPMSM is depicted in Figure 3. Both the stator and rotor of the CPMSM feature a conical shape. Given the technical requirements of a large diameter and short axial length for the shaftless propulsor, the CPMSM adopts a 24-pole/144-slot combination in accordance with the multi-polarization design criteria of PMSM.

Moreover, the elimination of the traditional propeller shaft system in the shaftless propulsor poses challenges to the installation of mechanical sensors, such as rotary transformers and photoelectric encoders. Therefore, a high-reliability and high-precision sensorless control strategy is typically employed to achieve accurate and real-time estimation of the motor’s rotational position, as well as speed and torque control [30]. To ensure excellent motor drive performance, the core magnetic flux density of the RDT motor is usually designed within the linear region of the material to guarantee its outstanding control performance. Hence, during the design process of the CPMSM, strict control of the magnetic flux density in the core will be implemented to ensure its operation in the linear region.

## 3. EMCM of CPMSM

### 3.1. Magnetic Circuit of CPMSM

**Figure 4:**
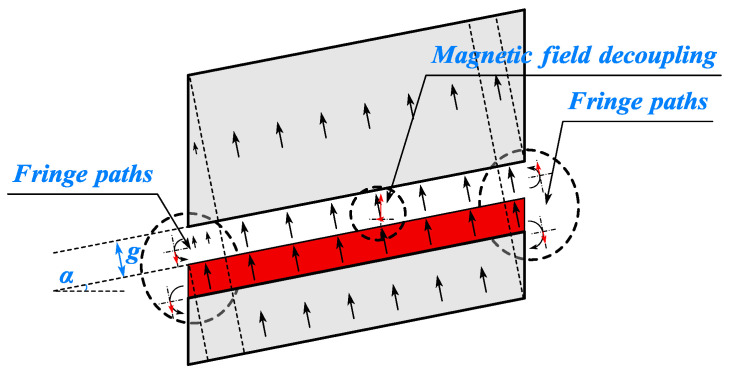
The distribution of magnetic fields of the CPMSM.

A 3D magnetic circuit model of the CPMSM is presented in Figure 4. In order to ensure the desirable electromagnetic characteristics of the CPMSM, the magnetization method for the permanent magnets in the CPMSM adopted in this paper is a parallel magnetization mode perpendicular to the conical surface.

**Figure 5:**
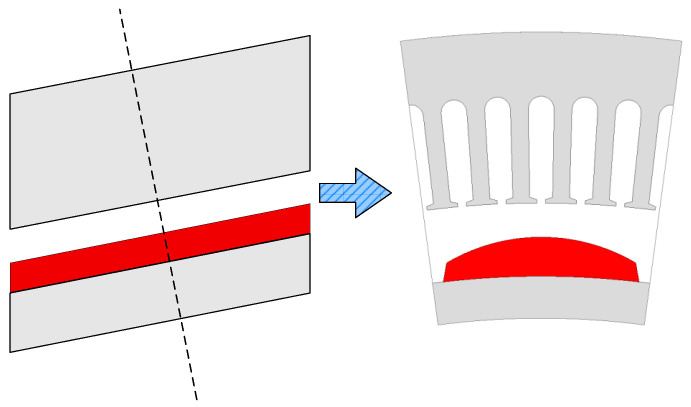
Simplified magnetic field model of CPMSM.

As illustrated in Figure 5, with this type of magnetization mode, except for the end magnetic circuit of the CPMSM, the 3D magnetic circuit model of the CPMSM can be simplified into a 2D magnetic circuit model that is vertically unfolded along the conical surface. This simplification facilitates the establishment of the equivalent magnetic circuit model of the CPMSM in subsequent research.

Based on the magnetic field decomposition principle and the reasonable assumption that the magnetic field is uniformly distributed along the conical surface of the CPMSM, the simplified magnetic field model of the CPMSM can be established. This model, derived from the 3D magnetic circuit model and simplified to a 2D model, serves as an important tool for analyzing and understanding the internal magnetic field distribution of the CPMSM. The following sections will elaborate on the EMCM of the CPMSM from four aspects: modeling of permanent magnets, modeling of CPMSM, modeling of end magnetic circuit, and modeling of nonlinear core materials.

### 3.2. Modeling of Permanent Magnets

This section discusses the modeling of permanent magnets (PMs) in a CPMSM by considering the impact of stator slots/teeth and rotor position on the magnetic flux distribution. The proposed EMCM is capable of analyzing and designing motors with any pole–slot combination. When the magnetic flux produced by the PMs does not link with the stator windings, this portion of the flux contributes nothing to the motor’s back electromotive force (EMF), and flux leakage occurs. Through proper modeling, the degree of flux leakage can be predicted, thereby optimizing the motor design. Specifically, the degree of flux leakage depends on the following factors: pole–slot combination, slot geometric features, and rotor position.

**Figure 6:**
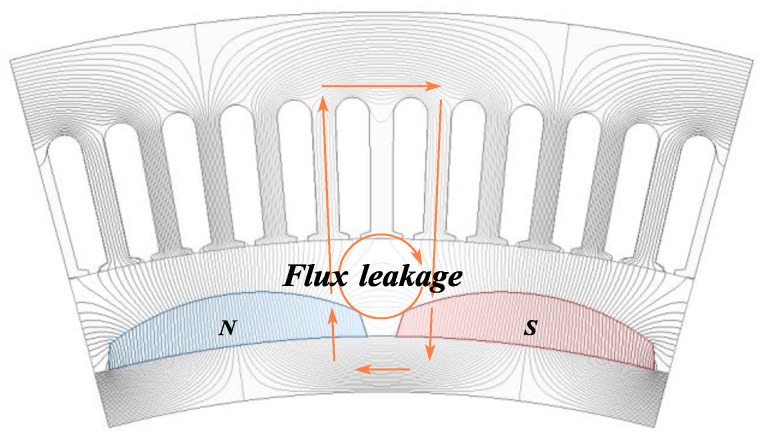
Flux cancelation mode.

This section analyzes the phenomenon of flux leakage through the following three modes:

Flux Cancelation Mode: As shown in Figure 6, in the flux cancelation mode, the leakage of magnetic flux is the most severe. Under this mode, the magnetic flux cancels out within the stator slots, resulting in the minimum contribution to the back EMF.

**Figure 7:**
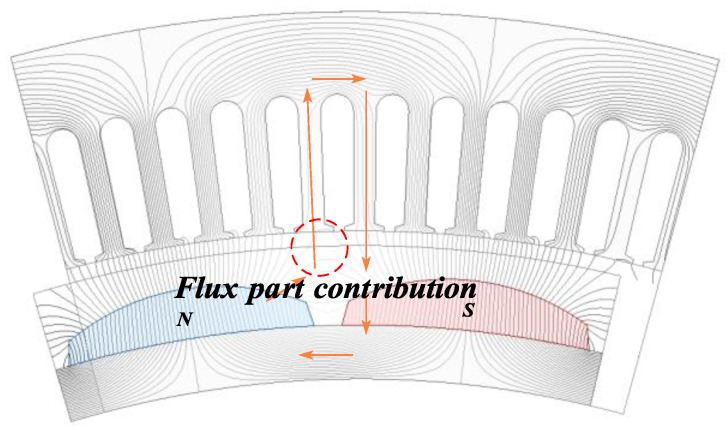
Flux partial contribution mode.

Flux Partial Contribution Mode: As shown in Figure 7, in the flux partial contribution mode, a portion of a magnetic pole of the PMs is located within the slot pitch. Due to the gap between the two magnetic poles, the flux contribution from the PMs is mitigated. The generated flux partially links with the stator windings, contributing to the back EMF, but not as significantly as in the full contribution mode.

**Figure 8:**
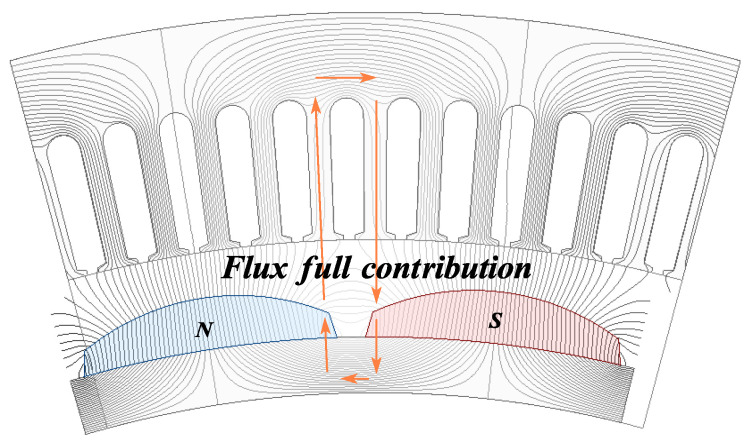
Flux full contribution mode.

Flux Full Contribution Mode: As shown in Figure 8, in the flux full contribution mode, the entire slot pitch is covered by a single pole. Under this mode, all the flux produced by the permanent magnet fully links with the stator windings, resulting in the maximum contribution to the back EMF.

**Figure 9:**
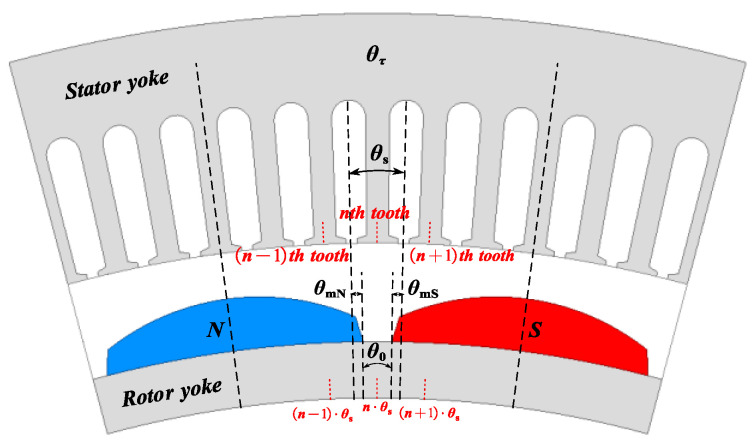
Features of PMs within a slot pitch.

Based on the preceding analysis, the PM model has been formulated. Taking into account a slot pitch *θ*_s_, it is posited that the PM’s contribution to the magnetomotive force (MMF) is directly proportional to the extent of the magnet within the range of *θ*_s_, as illustrated in Figure 9. For the slot pitch corresponding to the nth tooth, the angular gap between the two magnets is denoted by *θ*_0_. The angular extent of the N pole magnet is represented by *θ*_mN_, while *θ*_mS_ signifies the angular extent of the S pole magnet.

The effective angular span *θ*_eq_ of PMs within the slot pitch can be expressed as follows:

(1) $$\theta_{\mathrm{eq}}=C_{m}\cdot \theta_{s}$$

where $C_{m}$ denotes the effective flux coefficient of the stator tooth, defined as follows:

(2) $$C_{m}=\frac{\theta_{\mathrm{mN}}-\theta_{\mathrm{mS}}}{\theta_{s}},\;\;\;-1\leq C_{m}\leq 1$$

Here, $C_{m}$ varies with the stator tooth index n, reflecting the relative position between the stator and rotor. For instance:

$C_{m}=1$, stator tooth fully aligned with the N-pole and $C_{m}=-1$, stator tooth fully aligned with the S-pole, represent the flux full contribution mode, as illustrated in Figure 8.

$C_{m}=0$ corresponds to the flux cancelation mode, shown in Figure 6.

Intermediate $C_{m}$ values indicate flux partial contribution modes, depicted in Figure 7.

Consequently, regardless of the pole–slot combination, it is essential to compute $C_{m}$ for each stator tooth to quantify the flux linkage between PMs and stator windings. The generalized $C_{m}$ formulation of as a function of the n-th stator tooth is the following:

(3) $$C_{m}=C_{m}\left(n\right)$$

Equations (1)–(3) demonstrate the universality of this method across arbitrary pole–slot configurations. The determination follows this procedure:

1. Calculation of Stator Slot Pitch $\theta_{s}$:

(4) $$\theta_{s}=\frac{2\pi p}{Q}$$

where *Q* is the number of stator slots, and *p* is the number of pole pairs.

2. Calculation of Relative Tooth Pitch $\theta_{s_{1}}\left(n\right)$:

(5) $$\theta_{s_{1}}\left(n\right)=\left(n-1\right)\cdot \theta_{s},\;\;n=1,2,3,\cdots ,Q$$

To simplify the relative tooth pitch, $\theta_{s_{1}}\left(n\right)$, $\theta_{r}\left(n\right)$ is confined within an electrical angle range of 2π expressed as follows:

(6) $$\theta_{r}\left(n\right)=\mathrm{rem}\left\{\theta_{s_{1}}\left(n\right),2\pi \right\}$$

3. Identification of Pole Polarity Associated with Stator Tooth:

The sign of $\theta_{r}\left(n\right)$ in Equation (6) determines the pole polarity:

(7) $$\mathrm{sign}\left\{\theta_{r}(n)\right\}=\left\{\begin{array}{l} 1,\;\;\;\;\theta_{r}(n)⩽\pi \\ -1,\;\;otherwise. \end{array}\right.$$

4. Sorting of Relative Tooth Pitch ${\theta^{\prime }}_{s_{1}}\left(n\right)$:

Given the periodicity $\theta_{s_{1}}\left(n\right)=\theta_{s_{1}}\left(n\right)+2\pi$, the domain of $\theta_{s_{1}}\left(n\right)$ is defined as $\left[-\pi ,\pi \right]$.

(8) $${\theta^{\prime }}_{s_{1}}\left(n\right)=\mathrm{rem}\left\{\theta_{s_{1}}\left(n\right)+\pi ,2\pi \right\}-\pi$$

**Figure 10:**
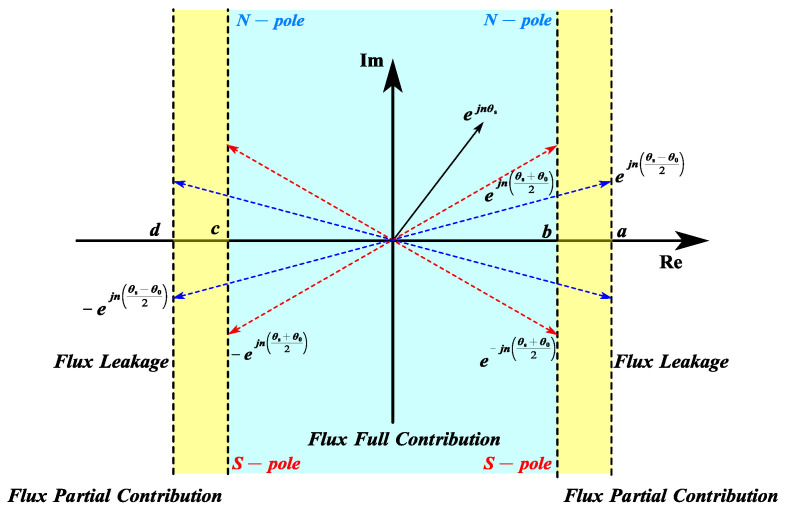
Modified slot pitch on polar coordinate.

When plotted on a polar coordinate system in Figure 10, ${\theta^{\prime }}_{s_{1}}\left(n\right)$ in the first/third quadrants and second/fourth quadrants are reciprocals. Thus, adding an arbitrary ±π in ${\theta^{\prime }}_{s_{1}}\left(n\right)$ does not result in any change in the calculation of $C_{m}$.

In Figure 10, the imaginary axis represents the polarity of the PM segment within the slot pitch. The real-axis projection indicates the operational state of $C_{m}$. For analytical convenience, ${\theta^{\prime }}_{s_{1}}\left(n\right)$ can be further constrained to $\left[-\pi /2,\pi /2\right]$.

(9) $${\theta^{\prime }}_{s_{1}}\left(n\right)=\left\{\begin{array}{l} {\theta^{\prime }}_{s_{1}}\left(n\right)-\pi ,\;{\theta^{\prime }}_{s_{1}}\left(n\right)\geq \pi \\ {\theta^{\prime }}_{s_{1}}\left(n\right)+\pi ,\;{\theta^{\prime }}_{s_{1}}\left(n\right)\leq -\pi \end{array}\right.$$

**Figure 11:**
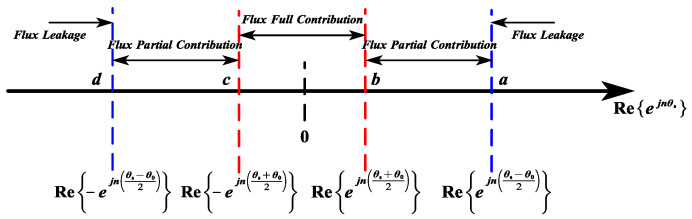
Boundaries of the modes on the real axis.

5. Boundary Conditions for Flux Partial Contribution Mode:

As illustrated in Figure 10 and Figure 11, the four boundary conditions (a, b, c, d) under flux partial contribution are defined by the following:

(10) a=Reejθs−θ02b=Reejθs+θ02c=Re−ejθs+θ02d=Re−ejθs−θ02

6. Computational Methodology:

Following the above steps, $C_{m}$ varies with stator tooth index *n* and rotor angular position:

(11) Cmn=signθrn,          Reejθs1′n≤bsignθrn⋅1−θs−θ0−2|θs1′n|2θs, b<Reejθs1′n<asignθrn⋅2|θs1′n|θs,      Reejθs1′n≥a

### 3.3. Modeling of CPMSM

The PM model was integrated into the developed CPMSM electromagnetic calculation model. Considering saturation effects, the magnetic flux on each stator tooth generated by the stator-winding current and PMs was calculated. During the iteration process, the permeability was updated based on the results of the previous round of calculations. For the CPMSM, a ring network was established, which used magnetic flux loops and node equations KCL and KVL to solve complex problems.

**Figure 12:**
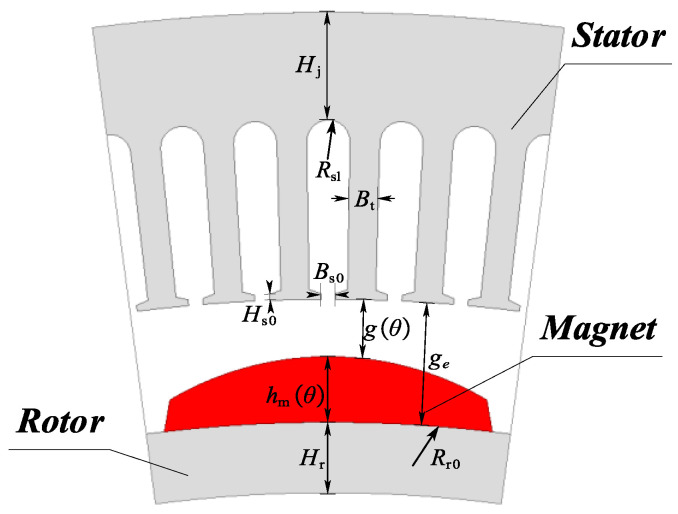
Geometry of CPMSM.

Magnetic Conductance Matrix: All components in the magnetic circuit are modeled based on the geometric structure and material properties of the motor. The definitions of the parameters shown in Figure 12 are based on the general geometric shape of the CPMSM slots, where *H*_r_ represents the rotor yoke thickness, *H*_j_ represents the stator yoke thickness, *B*_s0_ represents the slot-opening distance, *H*_s0_ represents the tooth shoulder height, *g*_e_ represents the mechanical air gap length, *B*_t_ represents the tooth width, *R*_sl_ represents the slot top radius, and *R*_r0_ represents the rotor outside diameter.

Other related parameters include the vacuum permeability *μ*_0_, the effective length of the motor *L*_ef_, the number of stator slots *Q*, and the relative permeability of the core *μ*_iron_.

**Figure 13:**
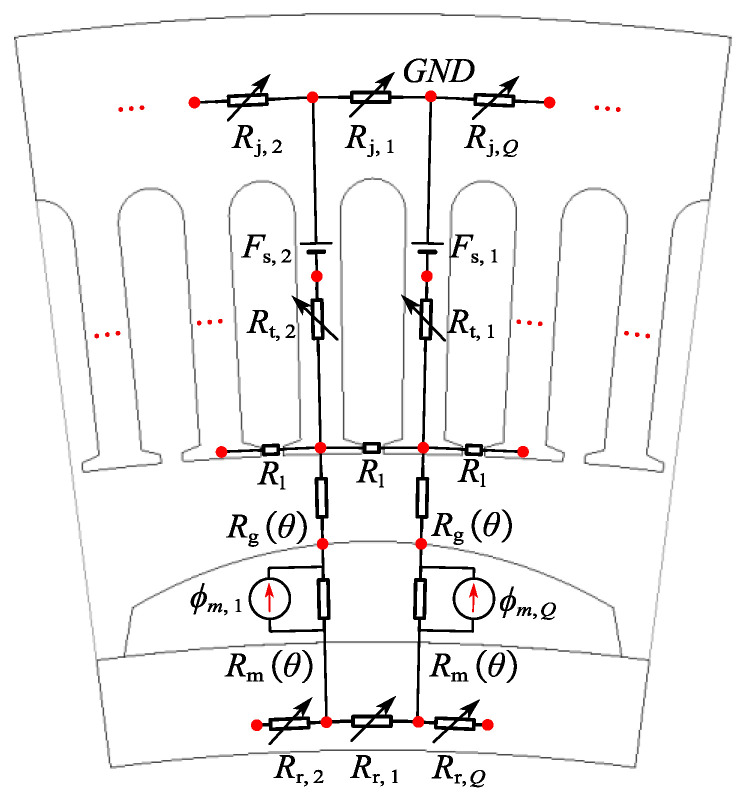
Modeling of CPMSM.

The following assumptions are made for the proposed CPMSM model:

1. As shown in Figure 13, the permeability of the stator and rotor cores is variable and represented by variable reluctance.
2. The flux passing through a stator tooth evenly traverses the air gap within one slot pitch angle.
3. Considering the impact of PM demagnetization on the electromagnetic performance of the CPMSM, the PM thickness $h_{m}\left(\theta \right)$ and mechanical air gap length are $g\left(\theta \right)$ defined as functions of θ.

As shown in Figure 12 and Figure 13, the air gap reluctance *R*_g_ is defined as follows:

(12) $$R_{g}=\frac{Q\cdot g\left(\theta \right)}{2\pi \cdot \mu_{0}\cdot \left[R_{r0}+h_{m}\left(\theta \right)+0.5\cdot g\left(\theta \right)\right]\cdot L_{\mathrm{ef}}}$$

The magnetic reluctance of the nth stator tooth $R_{t,n}$, the nth rotor yoke segment $R_{r,n}$, and the nth stator yoke segment $R_{j,n}$ are, respectively, represented as follows:

(13) $$\left\{\begin{array}{l} R_{t,n}(\mu_{\mathrm{iron},t,n})\\ =\frac{R_{\mathrm{sl}}-g(\theta )-R_{r0}-h_{m}(\theta )+0.5\cdot H_{j}}{\mu_{\mathrm{iron},t,n}\cdot \mu_{0}\cdot B_{t}\cdot L_{\mathrm{ef}}}\\ R_{r,n}(\mu_{\mathrm{iron},r,n})\\ =\frac{\pi \cdot (2R_{r0}-H_{r})}{Q\cdot \mu_{\mathrm{iron},t,n}\cdot \mu_{0}\cdot H_{r}\cdot L_{\mathrm{ef}}}+\frac{Q\cdot H_{r}}{\pi \cdot \mu_{\mathrm{iron},t,n}\cdot \left(R_{r0}-0.25H_{r}\right)\cdot L_{\mathrm{ef}}}\\ R_{j,n}(\mu_{\mathrm{iron},s,n})\\ =\frac{\pi \cdot (2R_{\mathrm{sl}}+H_{j})}{Q\cdot \mu_{\mathrm{iron},s,n}\cdot \mu_{0}\cdot H_{j}\cdot L_{\mathrm{ef}}} \end{array}\right.$$

where $\mu_{\mathrm{iron},t,n}$ is the relative permeability of the nth stator tooth, $\mu_{\mathrm{iron},r,n}$ is the relative permeability of the nth rotor yoke segment, and $\mu_{\mathrm{iron},s,n}$ is the relative permeability of the nth stator yoke segment. These parameters may change during the iteration process.

The magnetic reluctance of the PM $R_{m}$ is defined as

(14) $$R_{m}=\frac{Q\cdot h_{m}\left(\theta \right)}{2\pi \cdot \mu_{m}\cdot \mu_{0}\cdot \left[R_{r0}+0.5\cdot h_{m}\left(\theta \right)\right]\cdot L_{\mathrm{ef}}}$$

Among them, the residual magnetic flux of the PM $\phi_{m,n}$ and other undefined symbols will be explained in the following content.

**Figure 14:**
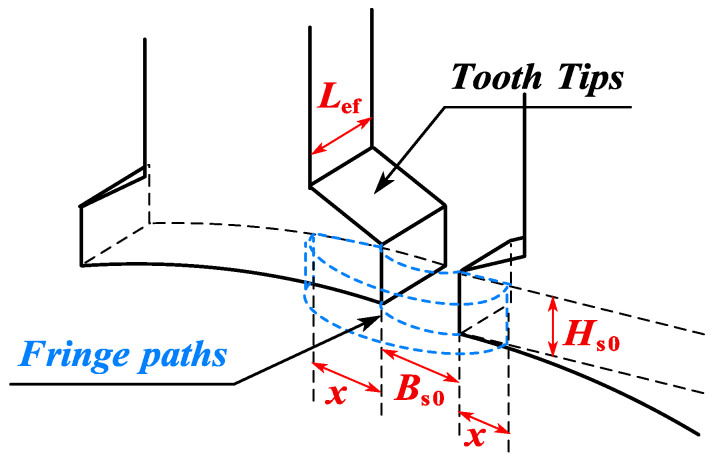
Flux pattern between tooth tips.

The flux leakage between two adjacent tooth tips is considered. As can be seen from Figure 14, the magnetic conductance of the slot between the two tooth tips (with a slot width of *B*_s0_) is affected by the edge effect, so it needs to be adjusted by a correction factor to improve the modeling accuracy.

For the magnetic flux distribution on the side of the tooth tip, as shown in Figure 14, the fringe path model of the transverse cross-section and its related parameters can be represented as [31]

(15) $$G_{\mathrm{fringe}}=\mu_{0}\cdot \frac{x\cdot H_{s0}}{0.17\cdot B_{s0}+0.14\cdot x}$$

where *x* represents the influence range of the edge magnetic conductance. The value of *x* is not limited by other geometric constraints, and the exact value chosen is not critical, since the contribution of the differential magnetic conductance will gradually decrease with the increase in x. In this case, the slot-opening length *B*_s0_ plays a dominant role in the contribution value of the edge magnetic conductance. Based on Equation (15), taking into account all four sides, the total leakage magnetic reluctance of the tooth tip air gap $R_{l}$ can be represented as

(16) $$R_{l}={\left[\frac{0.17\cdot B_{s0}+0.14\cdot x}{2\mu_{0}\cdot x\cdot \left(H_{s0}+L_{\mathrm{ef}}\right)}+\frac{B_{s0}}{\mu_{0}\cdot H_{s0}\cdot L_{\mathrm{ef}}}\right]}^{-1}$$

As shown in Figure 14, the leakage flux between two adjacent tooth tips is transmitted through the air and therefore is independent of the magnetic permeability of the tooth tip material. Although the tooth tip is prone to saturation and exhibits nonlinear characteristics, its impact on the overall performance of the motor is relatively small, so it is neglected here.

**Figure 15:**
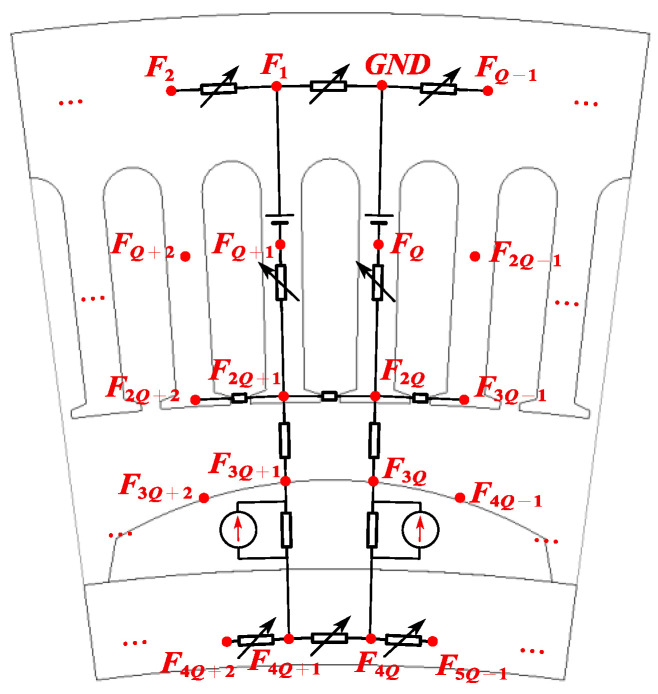
Definitions for the nodes.

The ampere-turns in a slot are represented by an MMF source. Figure 15 shows all the magnetic circuit components used in the magnetic network, where $F_{s,n}$ represents the MMF in the nth stator tooth. It should be noted that the magnetic reluctance in Equation (13) is numbered according to the magnetic circuit structure shown in Figure 15, and the definition of the nodes is represented in Figure 15.

Through the above derivation, CPMSMs with Q slots can be modeled. Once Q is determined, the corresponding CPMSM equivalent magnetic circuit model can be constructed. The EMCM is represented by a matrix, which can be solved using computer programming based on KCL and KVL.

Firstly, all parameters based on the motor’s geometric structure, material properties, and winding arrangement are arranged into a matrix of size $\left(6Q-1\right)\times \left(6Q-1\right)$ and defined as the magnetic conductance matrix *A*, as follows:

(17) $$A={\left[\begin{array}{ll} G & B\\ C & D \end{array}\right]}_{\left(6Q-1\right)\times \left(6Q-1\right)}$$

Among them, *G* is a matrix of size $\left(5Q-1\right)\times \left(5Q-1\right)$, and *G* is a function of *Q*. The matrix G can be represented as follows:

(18) $$G\left(Q\right)={\left[\begin{array}{lllll} G_{1} & G_{2} & G_{3} & G_{4} & G_{5}\\ G_{6} & G_{7} & G_{8} & G_{9} & G_{10}\\ G_{11} & G_{12} & G_{13} & G_{14} & G_{15}\\ G_{16} & G_{17} & G_{18} & G_{19} & G_{20}\\ G_{21} & G_{22} & G_{23} & G_{24} & G_{25} \end{array}\right]}_{\left(5Q-1\right)\times \left(5Q-1\right)}$$

The details of the submatrices (*G*_1_–*G*_25_) contained in matrix *G* are given in the Appendix A [Equations (A1)–(A10)], among them, all parameters can be obtained from Equations (12)–(16). It is noted that these matrices have different sizes and the combination of them will form matrix *G*.

The matrix *B* in Equation (17) is presented in the form of a Boolean function, and its sign is determined by the direction of the MMF source caused by the current in the winding. The matrix *B* can be represented by the following equation:

(19) $$B={\left[\begin{array}{l} B_{1}\\ B_{2}\\ B_{3} \end{array}\right]}_{\left(5Q-1\right)\times Q}$$

The submatrices in matrix *B* are given in the following equations:

(20) $$\left\{\begin{array}{l} B_{1}=\mathrm{diag}{\left[1\right]}_{Q\times Q}\\ B_{2}={\left[\begin{array}{llll} 0 & -1 & \cdots & 0\\ \vdots & & \ddots & \vdots \\ & & & 0\\ 0 & \cdots & & -1 \end{array}\right]}_{\left(Q-1\right)\times Q}\\ B_{3}=\mathrm{diag}{\left[0\right]}_{3Q\times Q} \end{array}\right.$$

Matrix *C* is the transpose of matrix *B*, and matrix *D* is a zero matrix.

(21) $$\left\{\begin{array}{l} C=B^{T}\\ D={\left[0\right]}_{Q\times Q} \end{array}\right.$$

In order to construct the equivalent magnetic circuit model of CPMSM, it is necessary to determine the relationship between the magnetomotive force source and the generated magnetic flux. Therefore, the matrix $\bar{x}$ is introduced, which is defined as follows:

(22) $$\bar{x}={\left[\begin{array}{l} F\\ \phi \end{array}\right]}_{\left(6Q-1\right)\times 1}$$

Among them, *F* represents the absolute value of the MMF at the nodes defined in the magnetic network in Figure 15, while ϕ represents the magnetic flux generated by *F*. The expressions for *F* and *Φ* are as follows:

(23) $$\left\{\begin{array}{l} F={\left[\begin{array}{llll} F_{1} & F_{2} & \cdots & F_{5Q-1} \end{array}\right]}_{\left(5Q-1\right)\times 1}^{T}\\ \phi ={\left[\begin{array}{llll} \phi_{1} & \phi_{2} & \cdots & \phi_{Q} \end{array}\right]}_{Q\times 1}^{T} \end{array}\right.$$

The elements in matrix ϕ represent the collection of magnetic fluxes associated with the nth stator tooth, such as stator tooth flux, air gap flux, stator yoke flux, and rotor yoke flux.

The magnitude of the magnetomotive force generated by the current in the winding and the permanent magnet can be defined by matrix *z*.

(24) $$z={\left[\begin{array}{llll} Z_{1} & Z_{2} & Z_{3} & Z_{4} \end{array}\right]}_{\left(6Q-1\right)\times 1}^{T}$$

where

(25) $$\left\{\begin{array}{l} Z_{1}={\left[0\right]}_{\left(3Q-1\right)\times 1}\\ Z_{2}=-Z_{3}={\left[\begin{array}{llll} \phi_{m,1} & \phi_{m,2} & \cdots & \phi_{m,Q} \end{array}\right]}_{Q\times 1}^{T} \end{array}\right.$$

wherein $\begin{array}{llll} \phi_{m,1} & \phi_{m,2} & \cdots & \phi_{m,Q} \end{array}$ is the magnetic flux source generated by the nth segment of the PM. For the nth stator tooth, the equivalent magnetic flux source generated by the PM can be represented as follows:

(26) $$\phi_{m,n}=B_{r}\cdot \tau_{\mathrm{mag}}\cdot C_{m}\left(n\right)\cdot \frac{2\pi \cdot \left[R_{r0}+0.5\cdot h_{m}\left(\theta \right)\right]\cdot L_{\mathrm{ef}}}{Q}$$

wherein $B_{r}$ represents the remanence of the PM, and $0\leq \tau_{\mathrm{mag}}\leq 1$ represents the ratio of the actual permanent magnet length to the pole pitch within a pole pitch. When $C_{m}=\pm 1$, the PM within the stator tooth becomes a completely independent source.

The MMF source $F_{s,n}$ generated by the winding current in the nth slot can be represented as Z_4_.

(27) $$Z_{4}={\left[\begin{array}{llll} F_{s,1} & F_{s,2} & \cdots & F_{s,Q} \end{array}\right]}_{Q\times 1}^{T}$$

Finally, the matrix $\bar{x}$ can be obtained using the following equation:

(28) $$\bar{x}=A^{-1}z$$

wherein $A^{-1}$ is the inverse matrix of matrix *A* in Equation (17).

An essential step in determining the working point of the material is to calculate the flux in various components of the motor. The flux in the nth stator tooth can be represented as follows:

(29) $$\left\{\begin{array}{l} \phi_{t,n}=\frac{F_{n+Q}-F_{n+2Q}}{R_{t,n}},\;n=1,2,3,\cdots ,Q-1\\ \phi_{t,Q}=\frac{-F_{2Q}}{R_{t,n}},\;\;\;\;n=Q \end{array}\right.$$

The air gap flux corresponding to the nth stator tooth can be represented as

(30) $$\phi_{g,n}=\frac{F_{n-1+2Q}-F_{n+3Q-1}}{R_{g}},n=1,2,3,\cdots ,Q$$

The stator yoke flux corresponding to the nth stator tooth can be represented as

(31) $$\left\{\begin{array}{l} \phi_{j,n}=\frac{F_{n}-F_{n+Q}}{R_{j,n}},\;n=1,2,3,\cdots ,Q-1\\ \phi_{j,Q}=\frac{F_{Q}}{R_{j,n}},\;\;\;\;n=Q \end{array}\right.$$

The rotor yoke flux corresponding to the nth stator tooth can be represented as

(32) $$\left\{\begin{array}{l} \phi_{r,n}=\frac{F_{n+4Q}-F_{n+4Q+1}}{R_{r,n}},\;n=1,2,3,\cdots ,Q-1\\ \phi_{r,Q}=\frac{F_{5Q-1}-F_{4Q}}{R_{r,n}},\;\;\;\;n=Q \end{array}\right.$$

For the PM, the magnetic flux density of the permanent magnet corresponding to the nth stator tooth can be represented as

(33) $$B_{m,n}=\frac{Q\cdot \left(F_{n-1+3Q}-F_{n+4Q-1}\right)}{2\pi \cdot R_{r0}\cdot L_{\mathrm{ef}}\cdot R_{m}},n=1,2,3,\cdots ,Q$$

The magnetic field intensity of the PM corresponding to the nth stator tooth can be represented as

(34) $$H_{m,n}=\frac{F_{n-1+3Q}-F_{n+4Q-1}}{h_{m}\left(\theta \right)},n=1,2,3,\cdots ,Q$$

In the proposed EMCM, the magnetic circuit network structure of the CPMSM is defined based on the number of slots *Q*. As a result, this method can be easily extended to CPMSM-type motors with any number of slots and poles, without the need to rebuild the magnetic circuit network structure for each specific case.

### 3.4. Nonlinearity of Ferromagnetic Materials

For ferromagnetic materials with nonlinear characteristics, their permeability varies with the operating point. Consequently, the permeability corresponding to the main components in the EMCM (the stator tooth, rotor yoke, and stator yoke) needs to be updated iteratively. The main steps are as follows:

1. EMCM Iteration Process: The nonlinear ferromagnetic materials in the EMCM are assigned a reasonable initial permeability value, and the calculation begins, as shown in Equation (13). After the first round of calculation, new conditions for the EMCM can be obtained, and the new flux in the main components can be derived through Equations (29), (31), and (32). Subsequently, the new flux density can be calculated accordingly.

(35) $$B_{\mathrm{new}}=\frac{\phi_{\mathrm{new}}}{S}$$

where $\phi_{\mathrm{new}}$ denotes the magnetic flux, and *S* is the cross-sectional area through which the magnetic flux $\phi_{\mathrm{new}}$ passes. The new magnetic field density $B_{\mathrm{new}}$ for the components in the EMCM is obtained from the operating point on the *B*–*H* curve. The new relative permeability $\mu_{\mathrm{new}}$ of the component can be expressed as follows:

(36) $$\mu_{\mathrm{new}}=\frac{\Delta B_{\mathrm{new}}}{\Delta H_{\mathrm{new}}}$$

Finally, the new relative permeability $\mu_{\mathrm{new}}$ of the component is applied to the EMCM to replace the initial values in the magnetic circuit components, thus completing one iteration. Usually, more iterations are needed to help the process converge.

**Figure 16:**
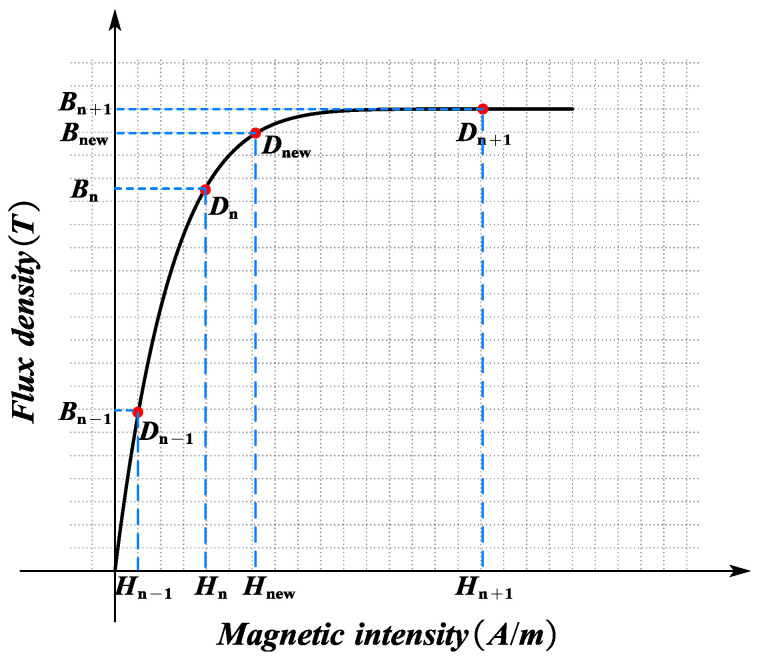
*B*–*H* curve of nonlinear ferromagnetic materials.

2. Permeability Update for Nonlinear Ferromagnetic Materials: A piecewise method is adopted to describe the *B*–*H* curve of nonlinear ferromagnetic materials, in order to take into account the impact of saturation effect on the electromagnetic performance of CPMSM. In this paper, an internal interpolation method is used to obtain the new permeability $\mu_{\mathrm{new}}$ in each iteration process. Consider the case where $B_{\mathrm{new}}$ is located between two consecutive operating points $D_{n},\;D_{n+1}$ on the hysteresis loop of the nonlinear material, as shown in Figure 16.

If there are enough data points on the *B*–*H* curve, the curve between $D_{n},\;D_{n+1}$ can be regarded as a straight line. Therefore, $H_{\mathrm{new}}$ can be expressed as

(37) $$H_{\mathrm{new}}=H_{n}+\left(H_{n+1}-H_{n}\right)\cdot \frac{B_{\mathrm{new}}-B_{n}}{B_{n+1}-B_{n}}$$

In order to prevent numerical divergence during the iteration process and improve the stability of the iteration, the substitution method (47) was improved by referring to the method of the previous value on the EMCM component during the iteration process. For example, before updating $\mu_{\mathrm{new}}$, the average value of $\mu_{\mathrm{new}}$ and the value from the previous iteration $\mu_{\mathrm{hold}}$ will be taken. The modified new relative permeability ${\overset{*}{\mu }}_{\mathrm{new}}$ can be expressed as follows:

(38) $${\overset{*}{\mu }}_{\mathrm{new}}=\frac{\mu_{\mathrm{new}}+\mu_{\mathrm{hold}}}{2}$$

**Figure 17:**
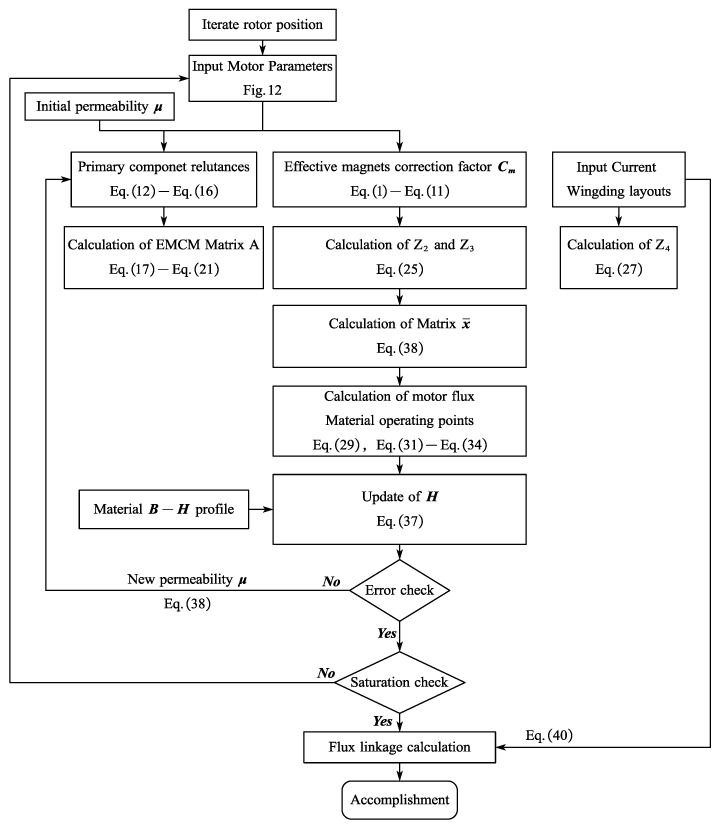
Iterating process of the EMCM approach of CPMSM.

To summarize the iteration process, Figure 17 provides a detailed illustration of the EMCM method for different rotor positions.

### 3.5. End Leakage Flux Coefficient of CPMSM

The end leakage flux of CPMSM is distinct from that of conventional PMSM due to the presence of a taper angle. For conventional PMSM, the magnetic field lines in the end air gap are much longer than those in other parts due to the end magnetic field, and some of the magnetic flux generated by the permanent magnets forms a closed loop with the rotor core at the end, resulting in a decay of the end magnetic flux density of PMSM.

**Figure 18:**
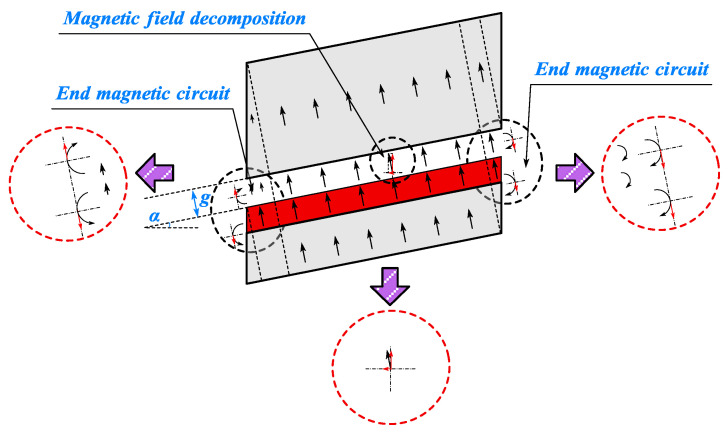
End magnetic circuit of CPMSM.

As shown in Figure 18, for CPMSM, the presence of a taper angle causes a difference in the thickness of the stator and rotor cores at the end. For the smaller-end section, the stator core thickness is greater than the rotor core thickness, so the magnetic field lines near the smaller-end section tend to be directed towards the stator core. For the larger-end section, the stator core thickness is less than the rotor core thickness, so the magnetic field lines near the larger-end section tend to be directed towards the rotor core.

According to the magnetic field decomposition principle, the magnetic field lines perpendicular to the conical surface in CPMSM are decomposed into radial and axial components. The radial air gap magnetic flux density at the end decays due to the influence of the end magnetic circuit, and the degree of decay depends on the saturation of the stator and rotor cores and the size of the taper angle of the CPMSM.

The axial air gap magnetic flux density at the end exhibits the following phenomena:

Although the overall end air gap magnetic flux density decays, the decay of the axial magnetic flux density depends on the difference between the decay of the end air gap magnetic flux density and the decay of the radial air gap magnetic flux density due to the presence of the taper angle, and it may even increase instead of decreasing.

Due to the taper angle of the CPMSM, the magnetic field lines near the larger-end section tend to be directed towards the rotor core, resulting in a phase reversal of the axial air gap magnetic flux density near the larger end section.

Based on the above inference, this section calculates the leakage flux coefficient of the permanent magnets in the CPMSM using the simplified equivalent magnetic circuit method to quantify the degree of end leakage flux in the CPMSM. For the topological structure of the slotted CPMSM, the influence of the slot width should be considered in the leakage flux coefficient.

**Figure 19:**
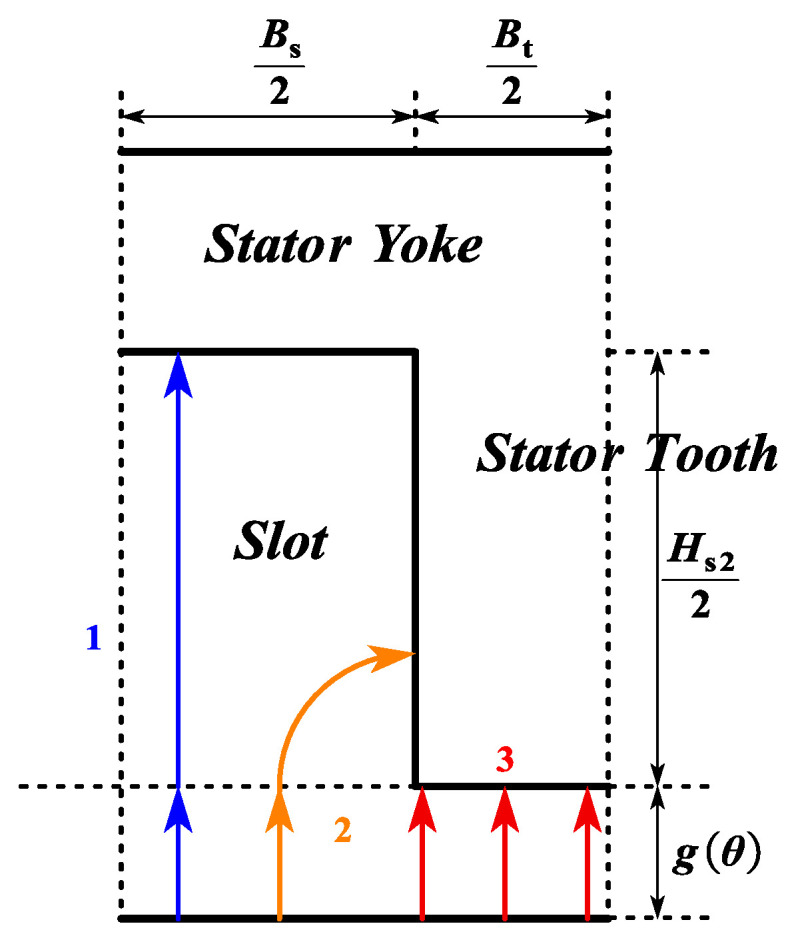
Path of permeances in calculating the Carter coefficient.

Therefore, the air gap length is corrected using the Carter coefficient. As shown in Figure 19, the different magnetic conductance paths (1, 2, 3) correspond to $\Lambda_{1}$, $\Lambda_{2}$, and $\Lambda_{3}$ in Equation (49), respectively. For $\Lambda_{4}$, it represents the air gap magnetic conductance in a slotless CPMSM.

(39) $$\left\{\begin{array}{l} \Lambda_{1}=\frac{\mu_{0}L_{\mathrm{ef}}}{2g\left(\theta \right)}\cdot B_{t},\Lambda_{2}=\frac{2\mu_{0}L_{\mathrm{ef}}}{\pi }\cdot \ln\left[1+\frac{\pi H_{s2}}{4g\left(\theta \right)}\right]\\ \Lambda_{3}=\frac{\mu_{0}L_{\mathrm{ef}}}{g\left(\theta \right)+H_{s2}}\cdot \left(\frac{B_{s}-H_{s2}}{2}\right),\Lambda_{4}=\frac{\mu_{0}L_{\mathrm{ef}}}{2g_{m}\left(\theta \right)}\cdot \left(B_{t}+B_{s}\right) \end{array}\right.$$

where $B_{t}$ and $B_{s}$ are the widths of the stator tooth and stator slot, respectively, and $H_{s2}$ is the height of the stator tooth. By adding $\Lambda_{1}$, $\Lambda_{2}$, and $\Lambda_{3}$, $\Lambda_{4}$ is obtained, and then the corrected air gap length $g_{m}\left(\theta \right)$ is calculated.

(40) $$g_{m}\left(\theta \right)=\frac{B_{t}+B_{s}}{\frac{B_{t}}{g\left(\theta \right)}+\frac{4}{\pi }\cdot \ln\left[1+\frac{\pi H_{s2}}{4g\left(\theta \right)}\right]+\frac{B_{s}-H_{s2}}{g\left(\theta \right)+H_{s2}}}$$

**Figure 20:**
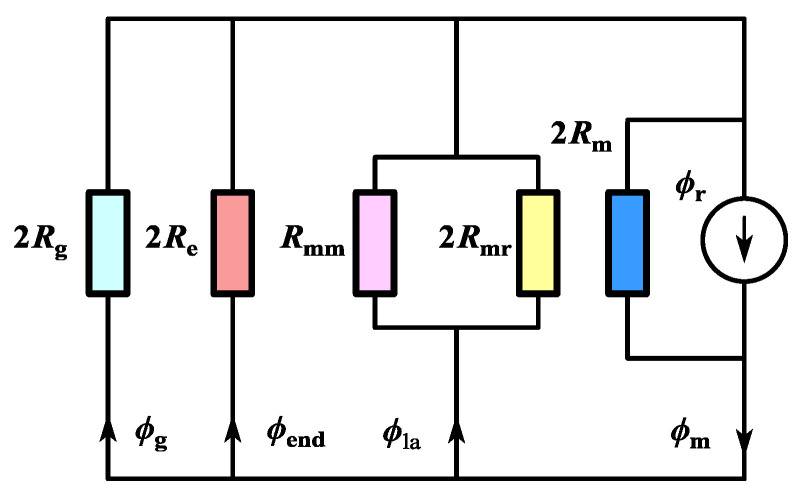
Magnetic circuit model of half pair of the PM.

Figure 20 presents the simplified end magnetic circuit model of the CPMSM. $\phi_{g}$, $\phi_{\mathrm{end}}$, and $\phi_{\mathrm{la}}$ represent the air gap flux, end leakage flux, and the leakage flux within the armature length, respectively.

**Figure 21:**
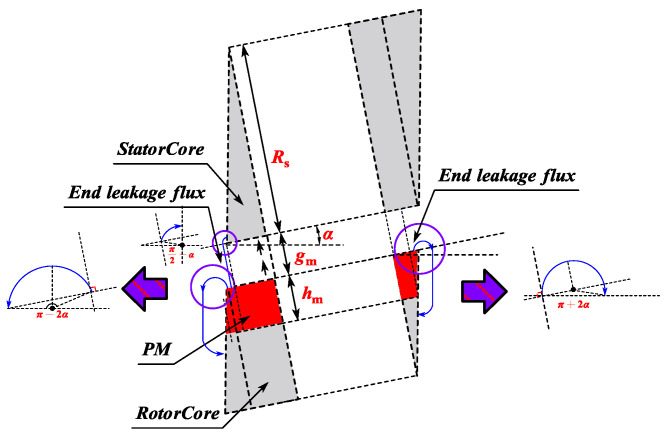
PM flux leakage path in the CPMSM end.

$\phi_{m}$ and $\phi_{r}$ represent the flux provided by the PM to the external magnetic circuit and the magnetic flux source generated by the permanent magnet, respectively. In Figure 20, the various magnetic reluctances can be calculated in the following way.

(41) $$\left\{\begin{array}{l} R_{\mathrm{mr}}={\left[\frac{\mu_{0}L_{\mathrm{ef}}}{\pi^{2}}\ln\frac{\pi g_{m}}{h_{m}}\right]}^{-1}\\ R_{\mathrm{mm}}={\left[\frac{\mu_{0}L_{\mathrm{ef}}}{\pi }\ln\frac{\pi g_{m}}{w}\right]}^{-1} \end{array}\right.$$

wherein *R*_g_ is the air gap reluctance without considering the edge effect, and *R*_mr_ and *R*_mm_ are the zigzag leakage flux reluctances caused by the PM and the rotor core, respectively. In this paper, it has been corrected by taking into account the end leakage flux of the CPMSM through *R*_end_, as shown in Figure 21.

In order to take into account the end leakage flux effect of the PM, the end leakage permeability can be calculated using the following method:

(42) $$\Lambda_{\mathrm{end}}=\int_{0}^{g_{m}}\frac{\mu_{0}w}{h_{m}+\left(3/2\right)\pi x}dx$$

With the flux division theory, the $\phi_{m}$ and $\phi_{\mathrm{end}}$ can be obtained as follows:

(43) $$\left\{\begin{array}{l} \phi_{m}=\frac{\nu +\sigma +\beta +2\gamma }{1+\nu +\sigma +\beta +2\gamma }\phi_{r}\\ \phi_{\mathrm{end}}=\frac{\sigma }{1+\nu +\sigma +\beta +2\gamma }\phi_{r} \end{array}\right.$$

The CPMSM leakage flux coefficient can be defined as

(44) $$k_{\mathrm{end}}=\frac{\phi_{r}}{\phi_{m}}=1-\frac{\nu +\beta +2\gamma }{\nu +\sigma +\beta +2\gamma }$$

(45) $$\left\{\begin{array}{l} \nu =\frac{R_{m}}{R_{\mathrm{mr}}},\sigma =\frac{R_{m}}{R_{e}}\\ \beta =\frac{R_{m}}{R_{\mathrm{mm}}},\gamma =\frac{R_{m}}{R_{g}} \end{array}\right.$$

When calculating the end magnetic field of the CPMSM, the flux matrix $\phi_{m}$ should be corrected using the CPMSM leakage coefficient $k_{\mathrm{end}}$. The corrected ${\overset{*}{\phi }}_{m}$ can be represented by the following equation:

(46) $${\overset{*}{\phi }}_{m}=\phi_{m}\cdot \left[1-\frac{\left(1-k_{\mathrm{end}}\right)\cdot \left(h_{m}+g\right)\cdot \tan\alpha }{L_{\mathrm{ef}}}\right]$$

where α is the taper angle of the CPMSM. By incorporating the corrected $\phi_{m}$ into the EMCM, the magnetic field calculation results of the CPMSM can be obtained.

## 4. Calculation and Optimization of Electromagnetic Performance for CPMSM

### 4.1. Electromagnetic Performance of CPMSM

**Figure 22:**
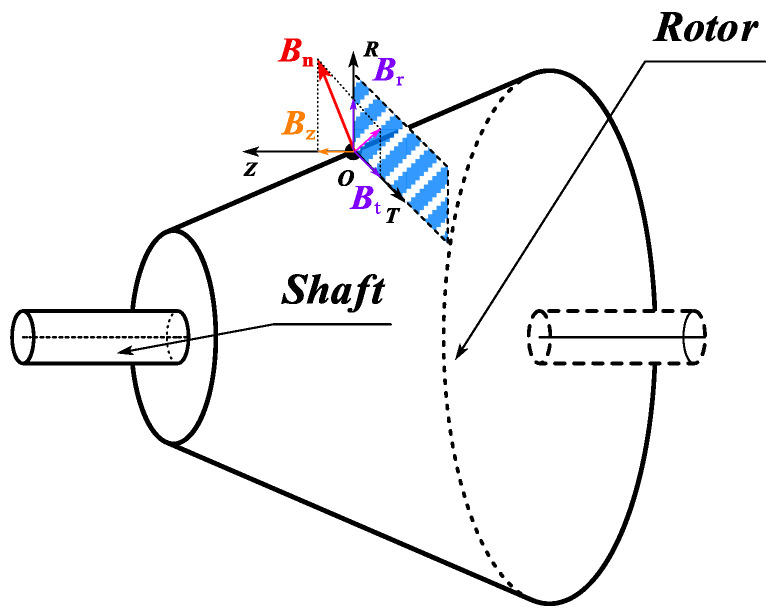
Magnetic field decomposition of CPMSM.

Through the above-mentioned magnetic field decomposition principle and EMCM, the air gap magnetic flux density results of the CPMSM can be easily obtained, as shown in Figure 22.

Among them, the normal magnetic flux density *B*_n_ and the tangential magnetic flux density *B*_t_ along the conical surface can both be obtained through the above-mentioned EMCM. As shown in Figure 21, *B*_n_ can be decomposed into the radial component *B*_r_ and the axial component *B*_z_. Considering the excellent driving and vibration performance requirements of CPMSM for shaftless propulsion, the core flux density under the rated load is generally selected in the linear working area. Therefore, it is assumed that the air gap magnetic field of CPMSM will not undergo nonlinear distortion. As a result, the expressions for the radial and axial components of the air gap magnetic field of CPMSM are as follows:

(47) $$\left\{\begin{array}{l} B_{r}=B_{n}\cdot \cos\alpha \\ B_{z}=B_{n}\cdot \sin\alpha \end{array}\right.$$

This paper calculates the electromagnetic characteristics of CPMSM using Maxwell’s tensor method, and the magnetic stress on the surface of the rotor core is

(48) $$\left\{\begin{array}{l} \sigma_{n}=\frac{B_{n}^{2}-B_{t}^{2}}{2\mu_{0}}\approx \frac{B_{n}^{2}}{2\mu_{0}}\\ \sigma_{t}=\frac{B_{n}\cdot B_{t}}{\mu_{0}} \end{array}\right.$$

**Figure 23:**
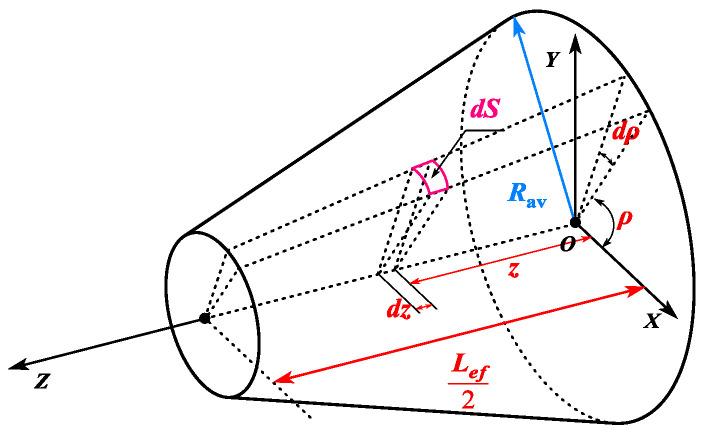
The topological structure of the CPMSM rotor.

As can be seen from Figure 23, the expression for *dS* is as follows:

(49) $$dS=\left(R_{\mathrm{av}}-z\cdot \tan\alpha \right)\cdot \frac{1}{\cos\alpha }dzd\rho$$

wherein *R*_av_ represents the average rotor radius of the CPMSM.

Consequently, the torque formula and the axial force formula of the CPMSM can be expressed as follows:

(50) $$\left\{\begin{array}{l} T=\frac{1}{\mu_{0}}\int_{0}^{2\pi }\int_{-\frac{L_{\mathrm{ef}}}{2}}^{\frac{L_{\mathrm{ef}}}{2}}B_{n}\left(\rho \right)\cdot B_{t}\left(\rho \right)\cdot {\left(R_{\mathrm{av}}-z\cdot \tan\alpha \right)}^{2}dzd\rho \\ F_{z}=\frac{1}{2\mu_{0}}\int_{0}^{2\pi }\int_{-\frac{L_{\mathrm{ef}}}{2}}^{\frac{L_{\mathrm{ef}}}{2}}B_{n}^{2}\left(\rho \right)\cdot \left(R_{\mathrm{av}}-z\cdot \tan\alpha \right)\cdot \tan\alpha dzd\rho \end{array}\right.$$

As can be seen from the above equation, the torque and axial force of the CPMSM are closely related to the taper angle, core length, average rotor radius, and the distribution of the air gap normal magnetic flux density.

### 4.2. Electromagnetic Performance Optimization of CPMSM

From the definition of *C*_m_ in the derived EMCM process (2), it can be seen that when the rotor PMs are arranged axially, and to ensure that the magnetic steel pole arc coefficient of each axial section of the CPMSM is consistent, the *C*_m_ value of each section of the CPMSM remains the same. This can greatly suppress the inter-polar leakage flux and enhance the performance of the CPMSM. By using the lumped-parameter method, the 3D magnetic field of the CPMSM can be completely transformed into a 2D magnetic field solution problem without any additional processing.

From Equations (47) and (50), it can be seen that in order to maintain the excellent electromagnetic performance of the CPMSM, such as low torque ripple and low axial force fluctuation, the taper angle of the CPMSM is determined by the shape of the shaftless propeller, and therefore the taper angle is not used as a basis for optimizing the electromagnetic performance of the CPMSM. As a result, according to the calculation results of the EMCM, the above optimization problem can be summarized as follows:

(51) $$\left\{\begin{array}{c} \max\;B_{n}\leq 0.6T\\ \max\;B_{t0}\leq 1.3T\\ \max\;B_{j}\leq 1.2T\\ \max\;B_{r}\leq 1.2T\\ \min\;ripple\left(T\right)\\ \min\;ripple\left(F_{z}\right)\\ \min\left(F_{z}\right)\\ T\geq T_{\mathrm{rate}} \end{array}\right.$$

By incorporating the above constraints into the EMCM of the CPMSM for solution, the required motor structural parameters of the CPMSM can be obtained.

## 5. FEA and Experimental Results Verification

### 5.1. CPMSM with FEA Verification

According to the above-derived results and the design requirements of the CPMSM, as shown in Table 1, a CPMSM prototype with a power rating of 10.5 kW and a taper angle of 6.7 degrees is designed using the proposed EMCM. The CPMSM adopted a combination of 24 poles and 144 slots. Comparisons between the EMCM and finite-element analysis (FEA) were made under both no-load and load conditions.

**Figure 24:**
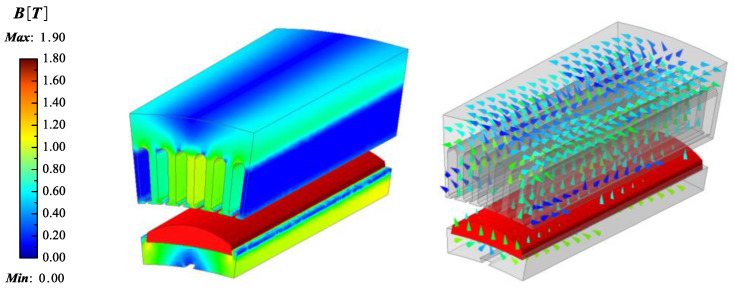
FEA model for CPMSM (no load).

**Figure 25:**
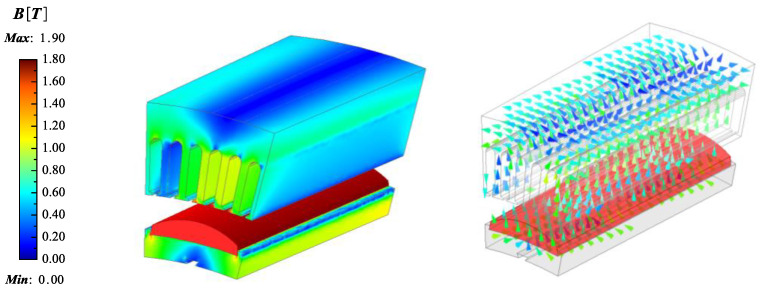
FEA model for CPMSM (on load).

This CPMSM can provide a rated torque of over 160 N·m at a speed of 630 rpm. The design results and material characteristics are detailed in Table 2, and the FEA model of the CPMSM is shown in Figure 24 and Figure 25.

In order to enhance the electromagnetic performance and vibration characteristics of the CPMSM, the CPMSM in this paper adopts a dual three-phase winding configuration.

To reduce the inter-polar leakage flux, a 10% pole pitch was used to separate the adjacent permanent magnets. Under these circumstances, a 10% pole pitch is equivalent to 1.5° mechanical angle, which can be used to calculate *C*_m_ in Equation (11). The stator core is made of 35WW270, the rotor core is made of 25Cr2Ni4MoV, and the permanent magnet is made of SmCo30H. The *B*–*H* curves of 35WW270 and 25Cr2Ni4MoV materials are generated through iterative interpolation in Figure 16. The back-EMF waveform is obtained through the derivative of the magnetic flux linkage, which is calculated via the magnetic flux in the stator teeth. Equation (40) is used to calculate the winding magnetic flux linkage.

**Figure 26:**
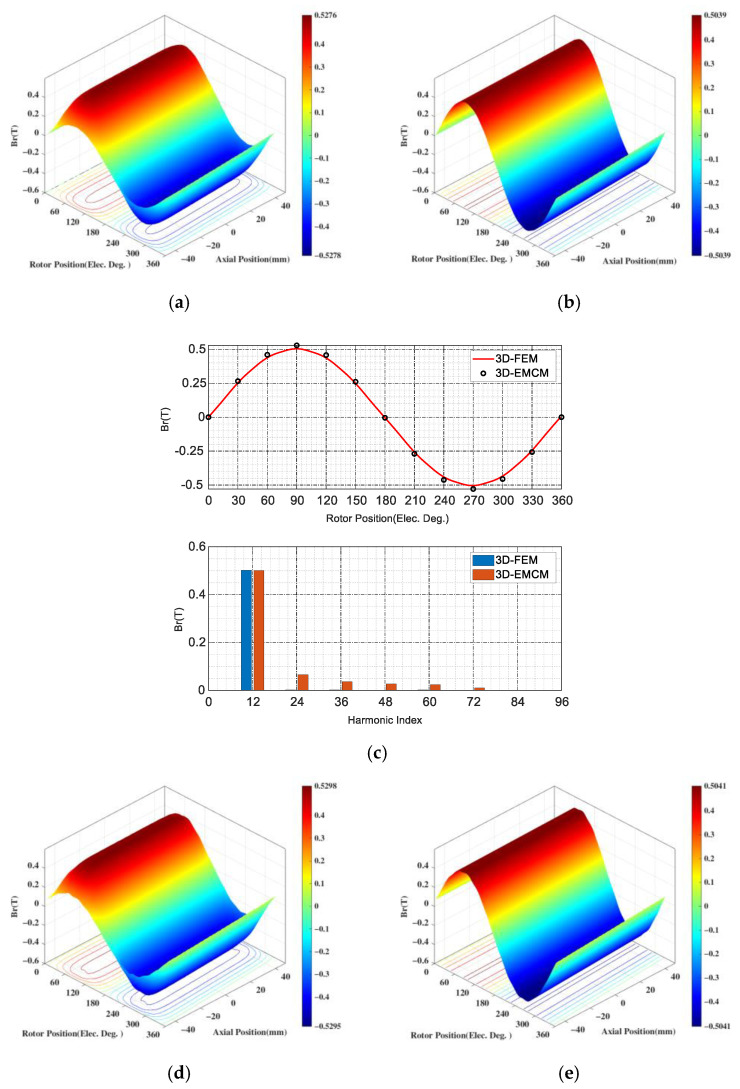

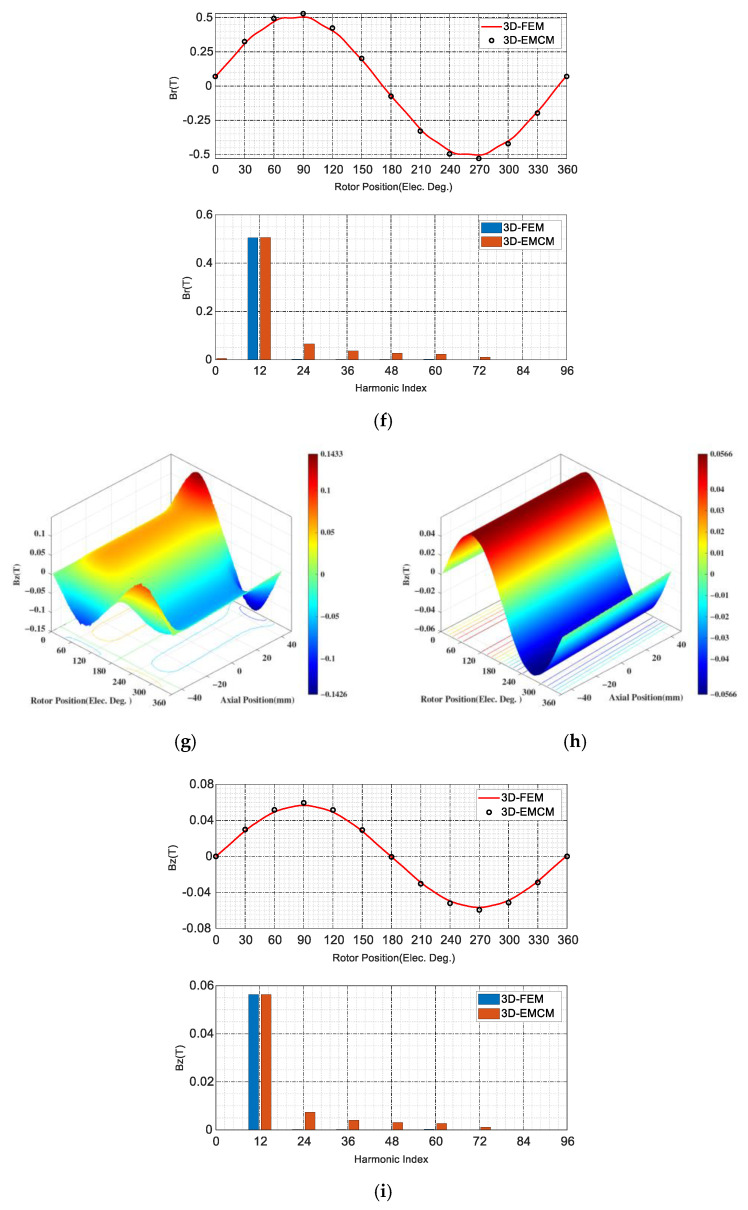

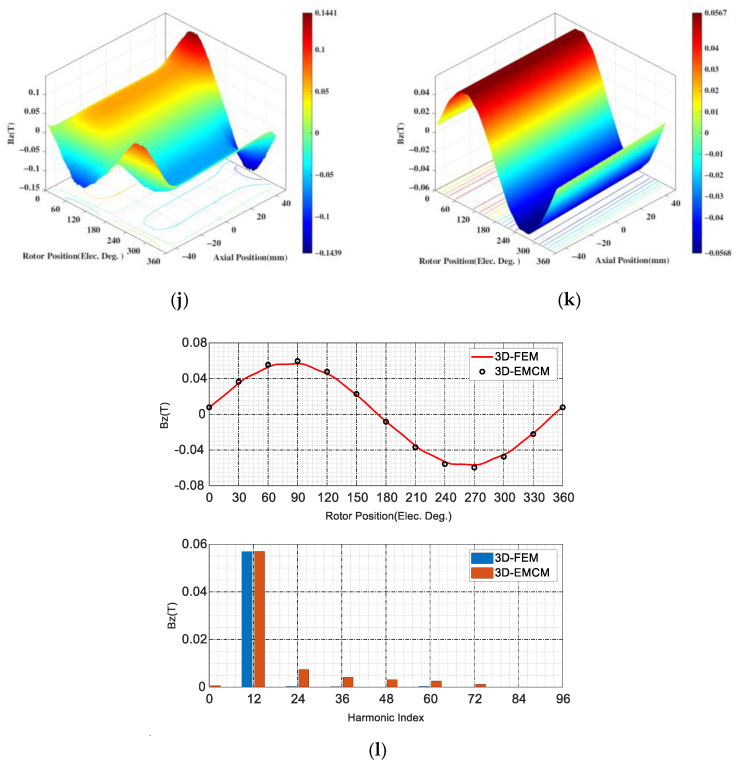
Air gap flux densities of CPMSM: (**a**) Radial components of 3D FEA (no load). (**b**) Radial components of 3D EMCM (no load). (**c**) Comparison of the radial air gap flux densities between 3D FEA and 3D EMCM (no load). (**d**) Radial components of 3D FEA (on load). (**e**) Radial components of 3D EMCM (on load). (**f**) Comparison of the radial air gap flux densities between 3D FEA and 3D EMCM (on load). (**g**) Axial components of 3D FEA (no load). (**h**) Axial components of 3D EMCM (no load). (**i**) Axial of the radial air gap flux densities between 3D FEA and 3D EMCM (no load). (**j**) Axial components of 3D FEA (on load). (**k**) Axial components of 3D EMCM (on load). (**l**) Comparison of the Axial air gap flux densities between 3D FEA and 3D EMCM (on load).

The comparison results between the developed equivalent magnetic circuit model and the FEA are shown in Figure 26, respectively.

The former employs the lumped-parameter method to account for the effects of magnetic circuit saturation and end-effects in the CPMSM, while the latter obtains the magnetic field results at each discrete location through distributed computation. It can be seen that the radial and axial magnetic flux densities obtained under no-load and load conditions are basically consistent between the two methods, with an error of less than 3%. Therefore, the validity of the CPMSM equivalent magnetic circuit model proposed in this paper is demonstrated.

### 5.2. CPMSM with Experiment Verification

**Figure 27:**
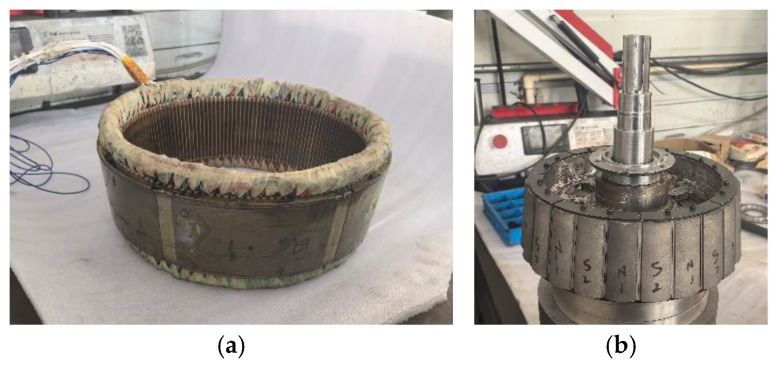
CPMSM prototype: (**a**) stator; (**b**) rotor.

Following the aforementioned design method, the CPMSM prototype corresponding to this paper was manufactured, and the stator and rotor structure of the CPMSM is shown in Figure 27.

**Figure 28:**
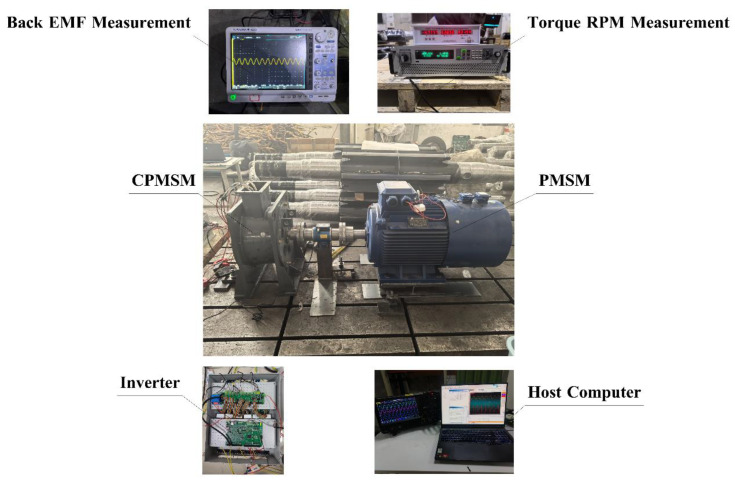
Test bench.

As shown in Figure 28, finally, the no-load back EMF of the prototype was measured under the rated speed, and the measured results were compared with those calculated by the EMCM and the FEA.

**Figure 29:**
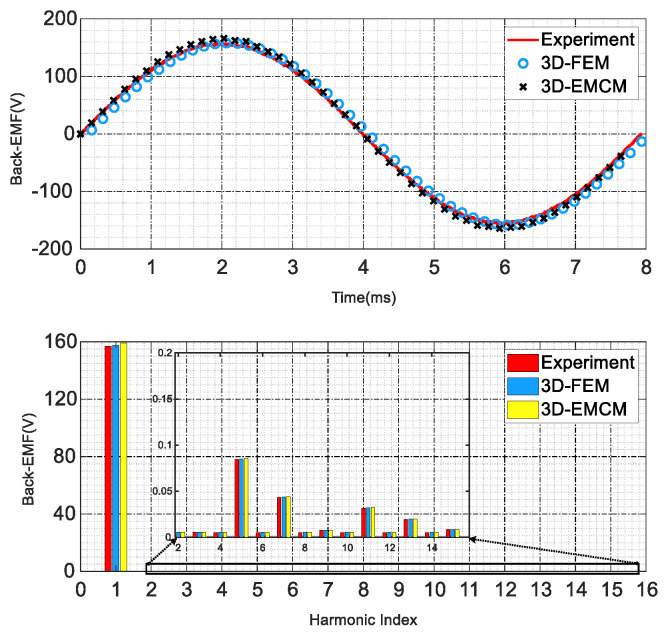
Comparison of the CPMSM back-EMF constants between FEA, EMCM, and experiment.

As shown in Figure 29, the no-load back EMF calculated through the EMCM and the finite element method is basically consistent with the experimental results. The error is within 3%. The experimental test results further prove the effectiveness of the EMCM proposed in this paper and provide a theoretical framework for the rapid design and performance evaluation of CPMSM for subsequent shaftless propulsion.

## 6. Conclusions

This paper proposes an equivalent magnetic circuit model for the design and analysis of CPMSM. First, the 3D magnetic field distribution of the CPMSM is equivalent to a 2D distribution through magnetic field analysis. Then, the model is based on the central angle of the CPMSM, including all the pole and slot information of the CPMSM, which can consider the impact of different PM shapes on the magnetic field performance of the CPMSM. The stator tooth effective magnetic flux coefficient *C*_m_ is set to account for the relationship of stator tooth flux variation during the operation of the CPMSM. Based on this, an iterative technique is proposed in this paper to take into account the nonlinearity of ferromagnetic materials. Finally, the model analyzes the end magnetic field of the CPMSM and calculates the impact of the end magnetic field on the CPMSM magnetic field through a simplified magnetic circuit model, which uses the lumped-parameter method to include the impact of the end magnetic field on the CPMSM magnetic field in the EMCM, thereby improving the calculation accuracy of the EMCM.

Finally, a 10.5 kW CPMSM with a taper angle of 6.7 degrees designed using the EMCM method was verified under different conditions (rated load and no load) by the finite element method and experiments, which verified the accuracy of the proposed model. The developed model is accurate enough to be used for the design of CPMSM without the need for time-consuming finite-element analysis.

## Figures and Tables

**Table 1:** CPMSM design requirements.

Parameters	Values
Rated speed (rpm)	630
Rated power (kW)	10.5
Rated torque (N·m)	160
Rated voltage (V)	200
Rated current (A)	30
Slot fill factor (%)	≤75
Current density (A/mm^2^)	≤8
Air gap length (mm)	6
Taper angle (degree)	6.7

**Table 2:** CPMSM design results.

Parameters	Values
Stator core outer diameter (mm)	365/387.08
Stator core inner diameter (mm)	305/327.08
Rotor core outer diameter (mm)	293/315.08
Rotor core inner diameter (mm)	264/286.08
Number of pole pairs	12
Number of slots	144
Maximum air gap flux density (T)	0.52
Maximum stator tooth flux density (T)	1.25
Maximum stator yoke flux density (T)	1.10
Maximum rotor yoke flux density (T)	1.15
Maximum magnet thickness (mm)	6.85
Permanent magnet material	SmCo30H
Slot opening (mm)	1.5
Stator tooth tip height (mm)	0.6
Stator tooth width (mm)	2.7
Stator tooth height (mm)	15.5
*R*_sl_ (mm)	176.8
*R*_r0_ (mm)	145.2
Stator yoke thickness (mm)	11.3
Rotor yoke thickness (mm)	7.3
Stator lamination thickness (mm)	0.35
Effective axial length of the motor (mm)	94

## Data Availability

Data are contained within the article.

## Appendix A

The submatrices in matrix *G* are given in the following Equations, including (A1)–(A10):

(A1) $$G_{1}={\left[\begin{array}{cccc} \frac{1}{R_{s,1}} & 0 & \cdots & 0\\ 0 & \frac{1}{R_{s,2}} & \cdots & \vdots \\ \vdots & \vdots & \ddots & 0\\ 0 & \cdots & 0 & \frac{1}{R_{s,Q}} \end{array}\right]}_{Q\times Q}$$

(A2) $$G_{2}={\left[\begin{array}{cccc} \frac{-1}{R_{s,1}} & 0 & \cdots & 0\\ 0 & \frac{-1}{R_{s,2}} & \cdots & \vdots \\ \vdots & \vdots & \ddots & \frac{-1}{R_{s,Q-1}}\\ 0 & \cdots & 0 & 0 \end{array}\right]}_{Q\times \left(Q-1\right)}$$

(A3) $$G_{7}={\left[\begin{array}{cccc} \frac{1}{R_{s,1}}+\frac{1}{R_{t,2}} & 0 & \cdots & 0\\ 0 & \frac{1}{R_{s,2}}+\frac{1}{R_{t,3}} & \cdots & \vdots \\ \vdots & \vdots & \ddots & 0\\ 0 & \cdots & 0 & \frac{1}{R_{s,Q-1}}+\frac{1}{R_{t,Q}} \end{array}\right]}_{\left(Q-1\right)\times \left(Q-1\right)}$$

(A4) $$G_{8}={\left[\begin{array}{ccccc} 0 & \frac{-1}{R_{t,2}} & 0 & \cdots & 0\\ 0 & 0 & \frac{-1}{R_{t,3}} & \cdots & 0\\ \vdots & 0 & 0 & \ddots & 0\\ 0 & \cdots & 0 & 0 & \frac{-1}{R_{t,Q}} \end{array}\right]}_{\left(Q-1\right)\times Q}$$

(A5) $$G_{14}={\left[\begin{array}{cccc} \frac{-1}{R_{g,1}} & 0 & \cdots & 0\\ 0 & \frac{-1}{R_{g,2}} & \cdots & \vdots \\ \vdots & \vdots & \ddots & 0\\ 0 & \cdots & 0 & \frac{-1}{R_{g,Q}} \end{array}\right]}_{Q\times Q}$$

(A6) $$G_{20}={\left[\begin{array}{cccc} \frac{-1}{R_{m,1}} & 0 & \cdots & 0\\ 0 & \frac{-1}{R_{m,2}} & \cdots & \vdots \\ \vdots & \vdots & \ddots & 0\\ 0 & \cdots & 0 & \frac{-1}{R_{m,Q}} \end{array}\right]}_{Q\times Q}$$

(A7) $$G_{13}={\left[\begin{array}{llll} \frac{2}{R_{l}}+\frac{1}{R_{t,1}}+\frac{1}{R_{g,1}} & \frac{-1}{R_{l}} & \cdots & \frac{-1}{R_{l}}\\ \vdots & & \ddots & \vdots \\ \frac{-1}{R_{l}} & 0 & \cdots & \frac{2}{R_{l}}+\frac{1}{R_{t,Q}}+\frac{1}{R_{g,Q}} \end{array}\right]}_{Q\times Q}$$

(A8) $$G_{19}={\left[\begin{array}{llll} \frac{1}{R_{m,1}}+\frac{1}{R_{g,1}} & 0 & \cdots & 0\\ \vdots & \ddots & & \vdots \\ 0 & \cdots & & \frac{1}{R_{m,Q}}+\frac{1}{R_{g,Q}} \end{array}\right]}_{Q\times Q}$$

(A9) $$G_{25}={\left[\begin{array}{llll} \frac{1}{R_{m,l}}+\frac{1}{R_{r,5}}+\frac{1}{R_{r,1}} & \frac{-1}{R_{r,1}} & \cdots & \frac{-1}{R_{r,Q}}\\ \vdots & & \ddots & \vdots \\ \frac{-1}{R_{r,Q}} & 0 & \cdots & \frac{1}{R_{m,Q}}+\frac{1}{R_{g,Q-1}}+\frac{1}{R_{g,Q}} \end{array}\right]}_{Q\times Q}$$

(A10) $$\left\{\begin{array}{l} G_{3}=G_{4}=G_{5}=G_{11}=G_{15}=G_{16}=G_{21}=G_{23}={\left[0\right]}_{Q\times Q}\\ G_{9}=G_{10}={\left[0\right]}_{\left(Q-1\right)\times Q}\\ G_{22}={\left[0\right]}_{Q\times \left(Q-1\right)}\\ G_{6}=G_{2}^{T}\\ G_{18}=G_{14}\\ G_{24}=G_{20} \end{array}\right.$$
